# Comparison of the Major Clinical Outcomes for the Use of Endeavor® and Resolute Integrity® Zotarolimus-Eluting Stents During a Three-Year Follow-up

**DOI:** 10.5334/gh.374

**Published:** 2020-02-06

**Authors:** Yong Hoon Kim, Ae-Young Her, Seung-Woon Rha, Byoung Geol Choi, Se Yeon Choi, Jae Kyeong Byun, Yoonjee Park, Dong Oh Kang, Won Young Jang, Woohyeun Kim, Cheol Ung Choi, Chang Gyu Park, Hong Seog Seo

**Affiliations:** 1 Division of Cardiology, Department of Internal Medicine, Kangwon National University School of Medicine, Chuncheon, KR; 2 Cardiovascular Center, Korea University Guro Hospital, Seoul, KR; 3 Department of Medicine, Korea University Graduate School, KR

**Keywords:** Clinical outcomes, Drug-eluting stent, Zotarolimus

## Abstract

**Background:**

Endeavor®-zotarolimus-eluting stent (E-ZES) was the first ZES to be developed, and Resolute integrity®-ZES (I-ZES) has been developed more recently. Comparative studies on long-term usage of these two ZESs have been rare.

**Objectives:**

The aim of this study was to compare the efficacy and safety of E-ZES and I-ZES during a long-term follow-up of patients who underwent percutaneous coronary intervention (PCI).

**Methods:**

A total of 767 patients who underwent PCI with E-ZES or I-ZES were eligible for this study. The primary endpoint was the occurrence of major adverse cardiac events (MACEs), defined as the composite of all-cause death, non-fatal myocardial infarction (MI), and any repeat revascularization. The secondary endpoint was stent thrombosis (ST).

**Results:**

After propensity score-matched (PSM) analysis, two PSM groups (193 pairs, n = 386, C-statistic = 0.824) were generated. During the 3-year follow-up period, the cumulative incidence of MACEs (hazard ratio [HR], 0.837; 95% confidence interval [CI], 0.464–1.508; p = 0.553) and ST (HR, 0.398; 95% CI, 0.077–2.052; p = 0.271) was similar for the E-ZES and I-ZES groups. Additionally, the cumulative incidences of all-cause death, cardiac death, non-fatal MI, and any repeat revascularization were not significantly different between the two groups.

**Conclusions:**

Although I-ZES utilizes a more advanced stent platform, stent design, and polymer system than E-ZES, both the ZESs showed comparable efficacy and safety during the 3-year follow-up period in this single-center, all-comers registry. However, further large-scaled, randomized, well-controlled trials with long-term follow-up are needed to verify these results.

Five kinds of zotarolimus-eluting stents, (ZESs) such as Endeavor® (E-ZES, Medtronic Cardiovascular, Santa Rosa, California, USA), Endeavor Sprint® (S-ZES), Endeavor Resolute® (R-ZES), Resolute Integrity® (I-ZES), and Resolute Onyx® are available in Korea. ZES was the third drug-eluting stent (DES) developed after the sirolimus-eluting stent (SES) and paclitaxel-eluting stent (PES). For E-ZES, the Driver® chromium-cobalt-nickel alloy coronary stent system is employed as the stent platform and it is designed using a modular technology. The polymer used in E-ZES was biomimetic phosphorylcholine (P)-polymer [1]. The platform used for I-ZES is the Integrity® chromium-cobalt-nickel based alloy stent; it is designed by continuous sinusoidal technology using the biocompatible BioLinx (B)-polymer coating system [2]. Thus, E-ZES and I-ZES have different stent platforms, stent design, and polymer system. In the P-polymer system, 75% of zotarolimus is release within 2 days whereas in the case of B-polymer system, the zotarolimus release is more delayed (50% and 85% zotarolimus is released at 7 and 60 days), and occurs for over 180 days after percutaneous coronary intervention PCI [3,4]. The B-polymer system was developed to decrease restenosis and maintain low rates of stent thrombosis (ST) through sustained release of zotarolimus for a longer duration [5]. Although several studies have reported the results of comparative analyses of the efficacy and safety of E-ZES and other DESs, such studies have been scarce for E-ZES and other types of ZESs [6,7,8,9]. In Korea, Resolute Onyx®-ZES was launched by Medtronic Korea and has been available only since March 2015. Although it is the most recently deployed ZES, patients with Resolute Onyx®-ZES were excluded from this study because of the short follow-up period. Thus, for the purpose of this study, E-ZES was the first developed and the oldest ZES, and I-ZES was considered the most recently developed ZES. These two stents were launched in Korea at a gap of about nine years. There have been very few studies comparing the major clinical outcomes of use of these two ZESs. The aim of the present study was to compare the efficacy and safety of E-ZES and I-ZES during the three-year follow-up of patients who underwent PCI.

## Material and Methods

### Study population

**Figure 1:**
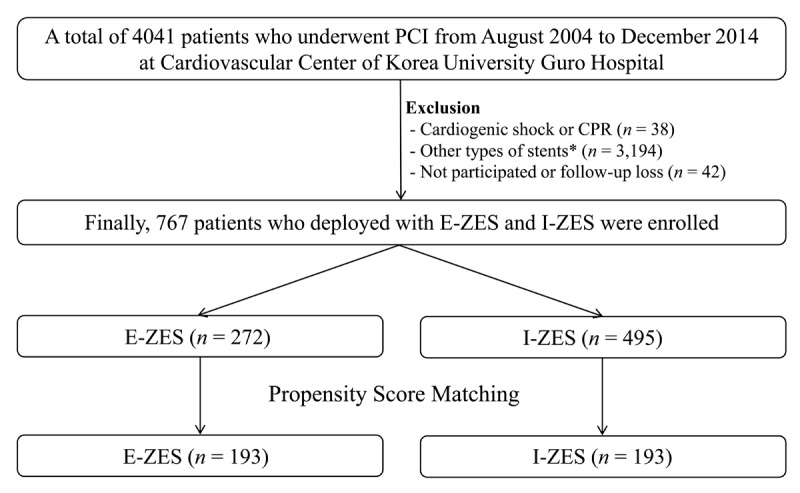
Flow chart.

This study was a single-center, retrospective, all-comers registry, designed to reflect the “real world” practice since 2004. A total of 4,041 patients who underwent PCI from January 2004 to December 2014 at the Cardiovascular Center of Korea University Guro Hospital, Seoul, South Korea were enrolled. Exclusion criteria were cardiogenic shock or cardiopulmonary resuscitation (*n* = 38), implantation of DESs other than E-ZES or I-ZES (*n* = 3194), and lost to follow-up or did not participate (*n* = 42). Finally, 767 patients with E-ZES (*n* = 272) or I-ZES (*n* = 495) were found to be eligible for this study. After a propensity score-matched (PSM) analysis, two baseline-matched groups (193 pairs, *n* = 386) were generated (Figure 1). This study was performed in accordance with the ethical standards laid down in the 1964 Declaration of Helsinki. The authors of this article certify that the information contained herein is true and correct, as reflected in the records of the Institutional Review Board; the Korea University Guro Hospital Institutional Review Board specifically approved the entire study. Data were collected by a trained study-coordinator using a standardized case report form.

### Study definitions and clinical follow-up

The primary endpoint of this study was the cumulative incidences of major adverse cardiac events (MACEs), defined as the composite of all-cause death, non-fatal myocardial infarction (MI), and any coronary repeat revascularization. Any coronary repeat revascularization was composed of target lesion revascularization (TLR), target vessel revascularization (TVR), and non-TVR. The secondary endpoint was the cumulative incidence of stent thrombosis (ST). All deaths were defined as cardiac or non-cardiac death. Non-fatal MI was defined as the presence of clinical symptoms, electrocardiographic changes, or findings of abnormal imaging of MI, combined with an increase in creatine kinase myocardial band fraction (CK-MB) above the upper normal limits, or an increase in troponin-T/troponin-I to greater than the 99th percentile of the upper normal limit [10]. The definitions of TLR, TVR, and non-TVR were as previously published [11]. ST (definite or probable) was defined as acute (0–24 hours), subacute (24 hours to 30 days), late (30 days to 1 year), and very late (>1 year), according to the onset time of ST [12]. All of cardiovascular risk factors and past medical histories were recorded based on the self-report furnished by the patients. The participants were required to visit the outpatient department of cardiology at the end of the first month, and then every 3 to 6 months after the index PCI procedure [13]. We were able to follow up on the clinical data of all the enrolled patients through face-to-face interviews at regular outpatient clinics, medical chart reviews, and telephonic contacts. A total of 767 patients finished their follow-up program.

### Percutaneous coronary intervention and medical treatment

Both a diagnostic coronary angiography (CAG) and PCI were done through either the femoral or the radial artery, after an administration of unfractionated heparin (70–100 IU/kg). The patient’s activated clotting time was maintained above 250 seconds during the procedure. All patients received a loading dose of 200–300 mg aspirin and 300–600 mg clopidogrel as the dual antiplatelet therapy (DAPT), and maintained with 100mg of aspirin and 75mg of clopidogrel. The use of cilostazol (Pletaal®, Otsuka Pharmaceutical Co., Tokyo, Japan) or platelet glycoprotein IIb/IIIa receptor blockers was left to the discretion of the individual operators. After stent implantation, DAPT (100-mg daily aspirin and 75mg daily clopidogrel) was prescribed for at least 12 months. During hospitalization, the enrolled patients took beneficial cardiovascular medications, including beta-blockers (BBs), angiotensin converting enzyme inhibitors (ACEIs) or angiotensin receptor blockers (ARBs), calcium channel blockers (CCBs), and lipid lowering agents. After discharge, the patients were encouraged to stay on the same medications they received during hospitalization.

### Statistical analysis

All data were processed with SPSS 20 (SPSS Inc, Chicago, IL, USA). For continuous variables, differences between the two groups were evaluated with the unpaired t-test or Mann-Whitney rank test. Data were expressed as mean ± standard deviations. For discrete variables, differences were expressed as counts and percentages, and analyzed with χ^2^ or Fisher’s exact test between the groups as appropriate. To adjust any baseline potential confounders, propensity score-matched (PSM) analysis was performed using the logistic regression model. We tested all the available variables that could be of potential relevance; gender (men), age, left ventricular ejection fraction (LVEF), stable angina, unstable angina, ST-segment elevation MI (STEMI), non-ST segment elevation MI (NSTEMI), coronary artery disease (CAD) risk factors, chronic kidney disease, laboratory findings, and post-PCI medications. Angiographic and procedural characteristics, such as target vessel, American College of Cardiology (ACC)/American Heart Association (AHA) B1/B2/C lesions, extent of CAD, treated chronic total obstructive (CTO) lesion, ostial lesion, diffuse long lesion (>30 mm), small vessel disease (≤2.25 mm), bifurcation lesion, heavy calcified lesion, mean total stent length, mean stent diameter, number of stents/patient, and total procedure time were also considered as covariates. The logistic model by which the propensity score (PS) was estimated showed good predictive value (C statistic = 0.824). Patients in the E-ZES group were then matched in a one-to-one manner to those in the I-ZES group according to propensity scores with the nearest available pair matching method. The subjects were matched with a caliper width equal to 0.2. The procedure yielded 193 well-matched pairs. To overcome the limitations of the PSM analysis, we also performed the multivariate analysis. We included only meaningful confounding covariates (*p* < 0.05 or having predictive values) during the multivariable Cox regression analysis, as shown in Table 3. Various clinical outcomes were estimated with the Kaplan-Meier method, and differences between the two groups were compared with the log-rank test. Proportional hazard models were used to compare the hazard ratio of E-ZES with the adjusted PS of I-ZES. For all analyses, a two-sided *p* value < 0.05 was considered statistically significant.

## Results

### Baseline clinical and angiographic characteristics

The baseline clinical and angiographic characteristics are shown in Table 1. Overall, in the total study population, the mean age (62.7 ± 10.5 years vs. 64.0 ± 11.2 years, *p* = 0.131) and sex distribution (men, 70.6% vs. 69.9%, *p* = 0.842) were similar between the two groups The numbers of patients with STEMI, dyslipidemia, previous PCI, routine angiographic follow-up, high sensitivity C-reactive protein, ACC/AHA type C lesion, and one-vessel disease was significantly higher in the E-ZES group than in the I-ZES group, as were the mean total stent length and stent diameter. In contrast, the degree of LVEF, the numbers of patients with diabetes, chronic kidney disease, targeted left circumflex coronary artery, multi-vessel disease, three-vessel disease, small vessel disease, and total procedure time were significantly higher in the I-ZES group than in the E-ZES group. However, all these differences were well-balanced after PSM.

**Table 1:** Baseline angiographic characteristics and post-PCI medications.

Variables	Total study population			Propensity-matched patients		

	E-ZES (*n* = 272)	I-ZES (*n* = 495)	*p*	E-ZES (*n* = 193)	I-ZES (*n* = 193)	*p*

Men, *n* (%)	192 (70.6)	346 (69.9)	0.842	136 (70.5)	134 (69.4)	0.824
Age (years)	62.7 ± 10.5	64.0 ± 11.2	0.131	63.2 ± 10.8	63.1 ± 11.7	0.882
LVEF (%)	51.0 ± 10.8	55.1 ± 8.6	<0.001	53.2 ± 9.8	54.1 ± 10.1	0.390
Stable angina, *n* (%)	60 (22.1)	133 (26.9)	0.142	45 (23.3)	58 (30.1)	0.135
Unstable angina, *n* (%)	101 (37.1)	168 (33.9)	0.375	79 (40.9)	63 (32.6)	0.091
ST segment elevation MI, *n* (%)	51 (18.8)	56 (11.3)	0.004	30 (15.5)	32 (16.6)	0.782
Non-ST segment elevation MI, *n* (%)	38 (14.0)	103 (20.8)	0.019	25 (13.0)	29 (15.0)	0.557
Hypertension, *n* (%)	161 (66.5)	326 (65.9)	0.848	128 (66.3)	133 (68.9)	0.587
Diabetes mellitus, *n* (%)	78 (28.7)	188 (38.0)	0.010	55 (28.5)	67 (34.7)	0.189
Dyslipidemia, *n* (%)	111 (40.8)	92 (18.6)	<0.001	59 (30.6)	60 (31.1)	0.912
Previous cerebrovascular accidents, *n* (%)	12 (4.4)	36 (7.3)	0.118	10 (5.2)	12 (6.2)	0.827
Previous MI, *n* (%)	1 (0.4)	1 (0.2)	0.667	1 (0.5)	0 (0.0)	0.317
Previous PCI, *n* (%)	9 (3.3)	0 (0.0)	<0.001	0 (0.0)	0 (0.0)	–
Peripheral vascular disease, *n* (%)	6 (2.2)	25 (5.1)	0.056	3 (1.6)	4 (2.1)	0.703
Chronic kidney disease, *n* (%)	9 (3.3)	34 (6.9)	0.040	8 (4.1)	8 (4.1)	1.000
Routine angiographic follow-up	190 (69.9)	176 (35.6)	<0.001	116 (60.1)	111 (57.5)	0.605
CK-MB (mg/dL), initial	31.8 ± 89.0	31.5 ± 80.0	0.969	28.6 ± 87.5	42.7 ± 100.5	0.159
Troponin T (ng/dL), initial	0.43 ± 1.41	0.55 ± 1.69	0.400	0.40 ± 1.48	0.74 ± 2.17	0.130
High sensitivity CRP (mg/dL)	10.9 ± 23.1	7.7 ± 11.1	0.015	7.2 ± 15.3	9.3 ± 14.8	0.185
Total cholesterol (mg/L)	169.5 ± 37.9	171.7 ± 45.8	0.529	168.0 ± 36.5	169.4 ± 47.0	0.747
Triglyceride (mg/L)	136.9 ± 75.5	147.9 ± 125.0	0.307	130.2 ± 68.5	144.6 ± 95.0	0.168
HDL cholesterol (mg/L)	45.6 ± 12.2	44.2 ± 11.0	0.190	46.1 ± 12.4	43.8 ± 11.1	0.126
LDL cholesterol (mg/L)	111.5 ± 34.2	108.3 ± 38.0	0.391	110.8 ± 34.2	106.9 ± 39.8	0.408
Serum creatinine (mg/L)	0.97 ± 0.56	1.06 ± 1.36	0.321	0.99 ± 0.65	0.99 ± 1.35	0.992
Serum glucose (mg/dL)	117.5 ± 43.2	124.6 ± 52.9	0.073	116.4 ± 45.0	122.2 ± 46.6	0.245
Hemoglobin A1_C_ (%)	6.4 ± 1.3	6.6 ± 1.3	0.108	6.4 ± 1.3	6.5 ± 1.3	0.462
Angiographic characteristics						
Targeted vessel						
Left anterior descending, *n* (%)	155 (57.0)	310 (62.6)	0.126	111 (57.5)	113 (58.5)	0.837
Left circumflex, *n* (%)	63 (23.2)	181 (36.6)	<0.001	46 (23.8)	53(27.5)	0.415
Right coronary artery, *n* (%)	111 (40.8)	172 (34.7)	0.096	76 (39.4)	74 (38.3)	0.835
Left main, *n* (%)	9 (3.3)	14 (2.8)	0.709	6 (3.1)	6 (3.1)	1.000
Ramus, *n* (%)	2 (0.7)	8 (1.6)	0.304	1 (0.5)	3 (1.6)	0.623
Multi-vessel disease (≥2 vessels)	60 (22.1)	156 (31.5)	0.005	42 (21.8)	48 (24.9)	0.470
ACC/AHA Lesion type						
Type B1, *n* (%)	16 (5.9)	30 (6.1)	0.921	12 (6.2)	8 (4.1)	0.492
Type B2, *n* (%)	46 (16.9)	124 (25.1)	0.009	32 (16.6)	45 (23.3)	0.098
Type C, *n* (%)	210 (77.2)	340 (68.7)	0.012	149 (77.2)	139 (72.0)	0.242
Extent of coronary artery disease, *n* (%)						
1-vessel	212 (77.9)	337 (68.1)	0.004	151 (78.2)	143 (74.1)	0.339
2-vessel	52 (19.1)	123 (24.8)	0.070	37 (19.2)	43 (22.3)	0.451
3-vessel	8 (2.9)	35 (7.1)	0.017	5 (2.6)	7 (3.6)	0.771
Treated CTO	11 (4.0)	37 (7.5)	0.061	6 (3.1)	14 (7.3)	0.106
Ostial lesion (≤5mm), *n* (%)	54 (19.9)	100 (20.2)	0.989	35 (18.1)	40 (20.7)	0.520
Diffuse long lesion (>30mm), *n* (%)	123 (45.2)	211 (42.6)	0.488	83 (43.5)	87 (45.1)	0.759
Small vessel disease (≤2.25), *n* (%)	15 (5.5)	77 (15.6)	<0.001	13 (6.7)	19 (9.8)	0.356
Bifurcation, *n* (%)	83 (30.5)	171 (34.5)	0.256	60 (31.1)	65 (33.7)	0.587
Heavy Calcification	36 (13.2)	80 (16.2)	0.279	26 (13.5)	30 (15.5)	0.563
Mean total stent length (mm)	23.7 ± 5.8	22.2 ± 6.6	0.003	23.3 ± 5.7	23.7 ± 7.1	0.532
Mean stent diameter (mm)	3.09 ± 0.45	2.98 ± 0.46	0.002	3.08 ± 0.45	3.06 ± 0.47	0.639
Number of stents/patient	1.31 ± 0.58	1.74 ± 1.05	<0.001	1.40 ± 0.72	1.48 ± 0.72	0.235
Total procedure time (minutes)	38.5 ± 29.5	44.7 ± 29.5	0.005	36.9 ± 25.4	41.8 ± 27.0	0.070
Post-PCI medications						
Aspirin, *n* (%)	253 (93.0)	461 (93.1)	0.951	182 (94.3)	180 (93.3)	0.673
Clopidogrel, *n* (%)	249 (91.5)	447 (90.3)	0.570	182 (94.3)	177 (91.7)	0.318
Cilostazol, *n* (%)	59 (21.7)	91 (18.4)	0.269	45 (23.3)	37 (19.2)	0.384
Beta blockers, *n* (%)	146 (53.7)	234 (47.3)	0.090	102 (52.8)	99 (51.3)	0.760
Calcium channel blockers, *n* (%)	96 (35.3)	187 (37.8)	0.495	63 (32.6)	70 (36.3)	0.453
ACEIs, *n* (%)	97 (35.7)	136 (27.5)	0.018	68 (35.2)	65 (33.7)	0.748
ARBs, *n* (%)	107 (39.3)	203 (41.0)	0.652	77 (39.9)	76 (39.4)	0.917
Diuretics, *n* (%)	70 (25.7)	90 (18.2)	0.014	45 (23.3)	44 (22.8)	0.904
Lipid lowering agents, *n* (%)	233 (85.7)	435 (87.9)	0.381	171 (88.6)	173 (89.6)	0.744

Values are mean ± SD or *n* (%). The *p* values for continuous data obtained from analysis of variance. The *p* values for categorical data obtained from chi-square test. E, Endeavor®; I, Resolute Integrity®; ZES, zotarolimus-eluting stent; LVEF, left ventricular ejection fraction; MI, myocardial infarction; CK-MB, creatine kinase myocardial band; CRP, C-reactive protein; HDL, high-density lipoprotein; LDL, low-density lipoprotein; ACC/AHA, American college of cardiology/American heart association; CTO, chronic total occlusive lesion. PCI, percutaneous coronary intervention; ACEIs, angiotensin converting enzyme inhibitors; ARBs, angiotensin receptor blockers.

### Post-percutaneous coronary intervention medications

The post-PCI medications for the two groups are also shown in Table 1. For all patients, the prescription rates of ACEIs (35.7% vs. 27.5%, *p* = 0.018) and diuretics (25.7% vs. 18.2%, *p* = 0.014) were significantly higher in the E-ZES group. The prescription of other medications (aspirin, clopidogrel, cilostazole, BBs, CCBs, ARBs, lipid lowering agents) was similar for the two groups.

### Clinical outcomes

**Figure 2:**
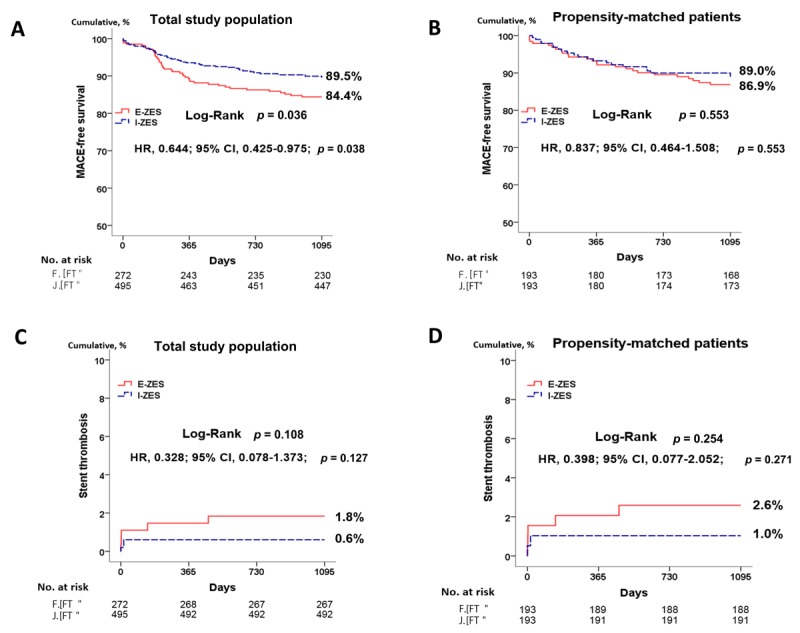
Kaplan-Meier curved analysis for MACE-free survival **(A, B)**, and stent thrombosis **(C, D)** at 3 years.

**Figure 3:**
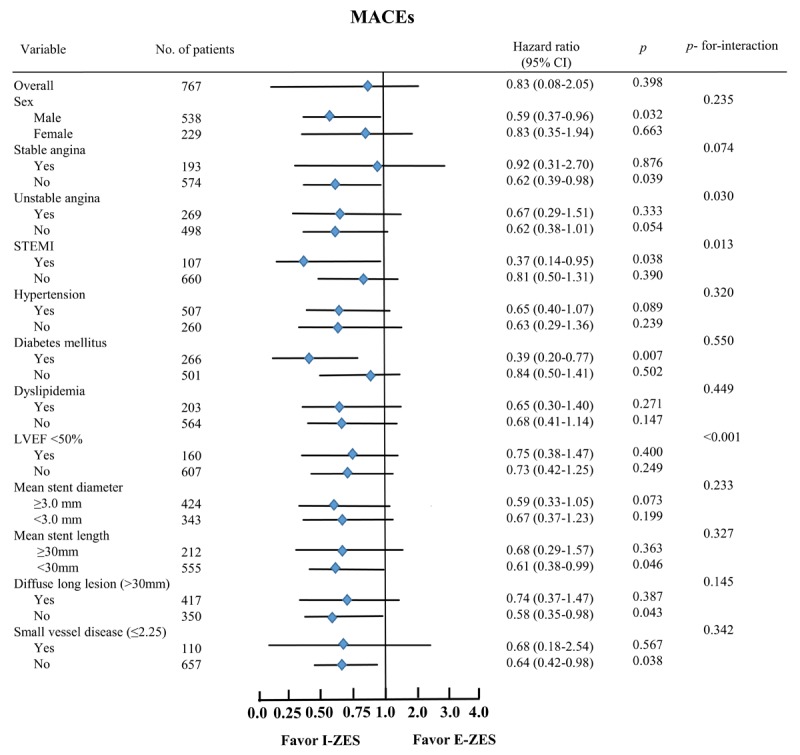
Subgroup analyses for MACEs.

The clinical outcomes at 30 days, 1 year, and 3 years for the E-ZES and I-ZES groups are presented in Table 2. For the one-month outcome, the cumulative incidence of MACEs was not significantly different between the two groups before PSM (1.5% vs. 1.4%, *p* = 0.950) and after PSM (2.1% vs. 0.5%, *p* = 0.177). At 1 year after the index PCI, the cumulative incidence of MACEs (10.7% vs. 6.5%, *p* = 0.040) was significantly higher in the E-ZES group compared to that in the I-ZES group before PSM. However, this difference was not statistically significant after PSM (6.7% vs. 6.7%, *p* = 1.000). The cumulative incidence of ST was similar for the two groups, regardless of PSM. At 3 years, the cumulative incidences of MACEs (15.4% vs. 9.7%, *p* = 0.018) and all-cause death (6.3% vs. 2.8%, *p* = 0.021) were significantly higher in the E-ZES group compared to those in the I-ZES group before PSM. After PSM, these differences disappeared (MACEs, 13.0% vs. 10.4%, *p* = 0.428; all-cause death, 7.8% vs. 3.6%, *p* = 0.079). The cumulative incidence of ST was comparable between the two groups before PSM (1.8% vs. 0.6%, *p* = 0.140) and after PSM (2.6% vs. 1.0%, *p* = 0.449). The results of Kaplan–Meier analysis for MACEs and ST at 3 years are shown in Figure 2. In the total study population, the cumulative incidence of MACE-free survival in the I-ZES group was higher than in the E-ZES group (HR, 0.644; 95% CI, 0.425–0.975; *p* = 0.038, Figure 2A). However, this difference between the two groups was statistically insignificant after the PSM analysis (HR, 0.837; 95% CI, 0.464–1.508; *p* = 0.553, Figure 2B). In the case of ST, the cumulative incidence of ST in the two groups was not significantly different before and after the PSM analysis. Additionally, the cumulative incidences of non-fatal MI, all-cause death, cardiac death, any repeat revascularization, TLR, TVR, and non-TVR were not significantly different between the two groups after PSM (Table 3). After multivariate analysis, the cumulative incidences of all major clinical outcomes were similar (in both groups) with those obtained after the PSM analysis (Table 3). The clinical outcome incidence rate according to the time to event variable is shown in Table 4. Even though the 3-year major clinical outcomes were similar in the two groups, the MACEs and mortality rates in the I-ZES group showed a tendency to be relatively higher than in the E-ZES group between the 30 days and 1-year follow-up periods. In both groups, most of the revascularization procedures were done between one month and 1.5 years after index PCI. A subgroup analysis for MACEs up to 3 years is shown in Figure 3. In cases of male (HR, 0.59; 95% CI, 0.37–0.96; *p* = 0.032), diabetes (HR, 0.39; 95% CI, 0.20–0.77; *p* = 0.007), less than 30 mm stent length (HR, 0.61; 95% CI, 0.38–0.99; *p* = 0.046), and less than 30 mm lesion length (HR, 0.58; 95% CI, 0.35–0.98; *p* = 0.043), I-ZES may be preferred over E-ZES to reduce the incidence of MACE after index PCI.

**Table 2:** Clinical outcomes at 30 days, 1 year, and 3 years.

Outcomes	Total study population				Propensity-matched patients		

	Total (*n* = 767)	E-ZES (*n* = 272)	I-ZES (*n* = 495)	*p*	E-ZES (*n* = 193)	I-ZES (*n* = 193)	*p*

30 days							
MACEs	11 (1.4)	4 (1.5)	7 (1.4)	0.950	4 (2.1)	1 (0.5)	0.177
All-cause death, *n* (%)	7 (0.9)	2 (0.7)	5 (1.0)	0.702	2 (1.0)	0 (0.0)	0.156
Cardiac death, *n* (%)	6 (0.8)	2 (0.7)	4 (0.8)	0.913	2 (1.0)	0 (0.0)	0.156
Non-fatal MI, *n* (%)	6 (0.8)	3 (1.1)	3 (0.6)	0.672	3 (1.6)	1 (0.5)	0.623
Any revascularization, *n* (%)	6 (0.8)	2 (0.7)	4 (0.8)	0.913	2 (1.0)	1 (0.5)	0.562
TLR, *n* (%)	5 (0.7)	2 (0.7)	3 (0.6)	0.832	2 (1.0)	1 (0.5)	0.562
TVR, *n* (%)	6 (0.8)	2 (0.7)	4 (0.8)	0.913	2 (1.0)	1 (0.5)	0.562
Non-TVR, *n* (%)	1 (0.1)	1 (0.4)	0 (0.0)	0.355	1 (0.5)	0 (0.0)	0.317
ST (definite or probable), *n* (%)							
Acute, *n* (%)	2 (0.3)	1 (0.4)	1 (0.2)	0.667	1 (0.5)	1 (0.5)	1.000
Subacute, *n* (%)	4 (0.5)	2 (0.7)	2 (0.4)	0.542	2 (1.0)	1 (0.5)	0.562
Total, *n* (%)	6 (0.8)	3 (1.1)	3 (0.6)	0.672	3 (1.6)	2 (1.0)	0.653
1-year							
MACEs, *n* (%)	61 (8.0)	29 (10.7)	32 (6.5)	0.040	13 (6.7)	13 (6.7)	1.000
All-cause death, *n* (%)	22 (2.9)	10 (3.7)	12 (2.4)	0.320	8 (4.1)	7 (3.6)	0.792
Cardiac death, *n* (%)	16 (2.1)	7 (2.6)	9 (1.8)	0.598	5 (2.6)	5 (2.6)	1.000
Non-fatal MI, *n* (%)	9 (1.2)	5 (1.8)	4 (0.8)	0.292	4 (2.1)	1 (0.5)	0.177
Any revascularization, *n* (%)	45 (5.9)	21 (7.7)	24 (4.8)	0.105	7 (3.6)	8 (4.1)	0.792
TLR, *n* (%)	30 (3.9)	17 (6.3)	13 (2.6)	0.013	6 (3.1)	5 (2.6)	0.760
TVR, *n* (%)	38 (5.0)	20 (7.4)	18 (3.6)	0.023	7 (3.6)	7 (3.6)	1.000
Non-TVR, *n* (%)	9 (1.2)	5 (1.8)	4 (0.8)	0.292	2 (1.0)	0 (0.0)	0.156
ST (definite or probable), *n* (%)							
Late (31–365 days)	1 (0.1)	1 (0.4)	0 (0.0)	0.177	1 (0.5)	0 (0.0)	0.317
Total (1–365 days)	7 (0.9)	4 (1.5)	3 (0.6)	0.253	4 (2.1)	2 (1.0)	0.685
3-year							
MACEs, *n* (%)	90 (11.7)	42 (15.4)	48 (9.7)	0.018	25 (13.0)	20 (10.4)	0.428
All-cause death, *n* (%)	31 (1.0)	17 (6.3)	14 (2.8)	0.021	15 (7.8)	7 (3.6)	0.079
Cardiac death, *n* (%)	19 (2.5)	10 (3.7)	9 (1.8)	0.113	8 (4.1)	5 (2.6)	0.574
Non-fatal MI, *n* (%)	20 (2.6)	11 (4.0)	9 (1.8)	0.095	10 (5.2)	4 (2.1)	0.172
Any revascularization, *n* (%)	59 (7.7)	25 (9.2)	34 (6.9)	0.248	10 (5.2)	12 (6.2)	0.661
TLR, *n* (%)	38 (5.0)	19 (7.0)	19 (3.8)	0.055	7 (3.6)	7 (3.6)	1.000
TVR, *n* (%)	54 (7.0)	24 (8.8)	30 (6.1)	0.152	10 (5.2)	12 (6.2)	0.661
Non-TVR, *n* (%)	11 (1.4)	5 (1.8)	6 (1.2)	0.532	2 (1.0)	1 (0.5)	0.562
ST (definite or probable), *n* (%)							
Very late (366–1095 days)	1 (0.1)	1 (0.4)	0 (0.0)	0.177	1 (0.5)	0 (0.0)	0.317
Total (1–1095 days)	8 (1.0)	5 (1.8)	3 (0.6)	0.140	5 (2.6)	2 (1.0)	0.449

Values are numbers and percentages. The *p* values for categorical data obtained from chi-square test. E, Endeavor®; I, Resolute Integrity®; ZES, zotarolimus-eluting stent; MACEs, major adverse cardiac events; MI, myocardial infarction; TLR, target lesion revascularization; TVR, target vessel revascularization.

**Table 3:** Three-Year Clinical Outcomes by Kaplan–Meier Curved Analysis and Cox-proportional Hazard Ratio Model Analysis.

Outcomes	Cumulative Events at 3 years (%)			Hazard Ratio (95% CI)	*p*

	E-ZES	I-ZES	Log Rank		

Total study population					
MACEs	42 (15.6)	48 (10.5)	0.036	0.644 (0.425–0.975)	0.038
All-cause death	17 (6.2)	14 (2.9)	0.034	0.474 (0.233–0.962)	0.039
Cardiac death	10 (3.7)	9 (1.8)	0.137	0.511 (0.207–1.259)	0.144
Non-fatal MI	11 (4.1)	9 (2.1)	0.110	0.494 (0.204–1.195)	0.118
Any revascularization	25 (9.5)	34 (7.4)	0.284	0.755 (0.450–1.266)	0.286
TLR	19 (7.1)	19 (4.2)	0.065	0.554 (0.293–1.048)	0.069
TVR	24 (9.0)	30 (6.7)	0.204	0.707 (0.413–1.211)	0.207
Non-TVR	5 (1.9)	6 (1.3)	0.495	0.663 (0.202–2.174)	0.498
Stent thrombosis	5 (1.8)	3 (0.6)	0.108	0.328 (0.078–1.373)	0.127
Propensity-matched patients					
MACEs	25 (13.1)	20 (11.0)	0.553	0.837 (0.464–1.508)	0.553
All-cause death	15 (7.8)	7 (3.6)	0.104	0.482 (0.196–1.183)	0.111
Cardiac death	8 (4.2)	5 (2.6)	0.432	0.641 (0.210–1.961)	0.436
Non-fatal MI	10 (5.3)	4 (2.3)	0.140	0.428 (0.134–1.367)	0.152
Any revascularization	10 (5.4)	12 (6.5)	0.622	1.235 (0.533–2.862)	0.623
TLR	7 (3.7)	7 (3.9)	0.991	1.006 (0.353–2.869)	0.991
TVR	10 (5.3)	12 (6.6)	0.598	1.253 (0.541–2.904)	0.599
Non-TVR	2 (1.1)	1 (0.6)	0.578	0.512 (0.046–5.653)	0.585
Stent thrombosis	5 (2.6)	2 (1.0)	0.254	0.398 (0.077–2.052)	0.271
Multivariate analysis*					
MACEs	42 (15.6)	48 (10.5)	0.036	0.943 (0.559–1.563)	0.820
All-cause death	17 (6.2)	14 (2.9)	0.034	0.433 (0.175–1.070)	0.070
Cardiac death	10 (3.7)	9 (1.8)	0.137	0.597 (0.191–1.863)	0.374
Non-fatal MI	11 (4.1)	9 (2.1)	0.110	0.481 (0.164–1.408)	0.182
Any revascularization	25 (9.5)	34 (7.4)	0.284	1.374 (0.721–2.614)	0.334
TLR	19 (7.1)	19 (4.2)	0.065	1.138 (0.515–2.512)	0.749
TVR	24 (9.0)	30 (6.7)	0.204	1.322 (0.677–2.584)	0.414
Non-TVR	2 (1.1)	1 (0.6)	0.578	0.697 (0.163–2.984)	0.627
Stent thrombosis	5 (2.6)	2 (1.0)	0.254	0.536 (0.103–2.788)	0.459

* Adjusted by age, men, LVEF, STEMI, NSTEMI, diabetes, dyslipidemia, previous history of PCI, CKD, routine angiographic follow-up, serum level of hs-CRP, LCx (targeted vessel), multi-vessel disease, ACC/AHA type B2/C lesion, 1-vessel disease, 3-vessel disease, small vessel disease, mean total stent length, mean stent diameter, number of stents/patient, total procedure time, ACEIs, diuretics. E, Endeavor®; I, Resolute Integrity®; ZES, zotarolimus-eluting stent; CI, confidence interval; MACEs, major adverse cardiac events; MI, myocardial infarction; TLR, target lesion revascularization; TVR, target vessel revascularization; CKD, chronic kidney disease; hs-CRP, high-sensitivity C-reactive protein; LCx, left circumflex artery; ACC/AHA, American College of Cardiology/American Heart Association; PCI, percutaneous coronary intervention; ACEIs, angiotensin converting enzyme inhibitors.

**Table 4:** Outcome incidence rates according to time to event.

	MACEs		All-cause death		Cardiac death		Non-fatal MI		Any revascularization		TLR		TVR		Non-TVR		Stent thrombosis	

	E-ZES	I-ZES	E-ZES	I-ZES	E-ZES	I-ZES	E-ZES	I-ZES	E-ZES	I-ZES	E-ZES	I-ZES	E-ZES	I-ZES	E-ZES	I-ZES	E-ZES	I-ZES

Total study population (*n* = 767) E-ZES (*n* = 272), I-ZES (*n* = 495)																		
Number of events, *n* (%)	42	48	17	14	10	9	11	9	25	34	19	19	24	30	5	6	5	3
Number of events/patients	0.15	0.10	0.06	0.03	0.04	0.02	0.04	0.02	0.09	0.07	0.07	0.04	0.09	0.06	0.02	0.001	0.02	0.01
Time to event (days), *n* (%)																		
1–7	3 (7)	3 (6)	1 (6)	2 (14)	1 (10)	2 (22)	2 (18)	1 (11)	2 (8)	0	2 (11)	0	2 (8)	0	1 (20)	0	3 (60)	1 (33)
8–30	1 (2)	4 (8)	1 (6)	3 (21)	1 (10)	2 (22)	1 (9)	2 (22)	0	4 (12)	0	3 (16)	0	4 (13)	0	0	0	2 (67)
31–180	9 (21)	11 (23)	4 (24)	4 (29)	3 (30)	2 (22)	2 (18)	1 (11)	7 (28)	8 (24)	5 (26)	5 (26)	7 (29)	7 (23)	0	1 (17)	1 (20)	0
181–365	16 (38)	14 (29)	4 (24)	3 (21)	2 (20)	3 (33)	0	0	12 (48)	12 (35)	10 (53)	5 (26)	11 (46)	7 (23)	4 (80)	3 (50)	0	0
366–548	5 (12)	5 (10)	3 (18)	1 (7)	1 (10)	0	2 (18)	2 (22)	2 (8)	3 (9)	2 (11)	2 (11)	2 (8)	3 (10)	0	0	1 (20)	0
549–730	3 (7)	7 (15)	1 (6)	1 (7)	0	0	1 (9)	1 (11)	0	5 (15)	0	2 (11)	0	5 (17)	0	2 (33)	0	0
731–913	3 (7)	2 (4)	2 (12)	0	2 (20)	0	3 (27)	2 (22)	1 (4)	1 (3)	0	1 (5)	1 (4)	3 (10)	0	0	0	0
914–1095	2 (5)	2 (4)	0	0	0	0	0	0	1 (4)	1 (3)	0	1 (5)	1 (4)	1 (3)	0	0	0	0

Propensity-matched patients (*n* = 386) E-ZES (*n* = 193), I-ZES (*n* = 193)																		
Number of events, *n* (%)	25	20	15	7	8	5	10	4	10	12	7	7	10	12	2	1	5	2
Number of events/patients	0.13	0.10	0.08	0.04	0.04	0.03	0.05	0.02	0.05	0.06	0.04	0.04	0.05	0.06	0.01	0.01	0.03	0.01
Time to event (days), *n* (%)																		
1–7	3 (12)	0	1 (7)	0	1 (13)	0	2 (20)	0	2 (20)	0	2 (29)	0	2 (20)	0	1 (50)	0	3 (60)	1 (33)
8–30	1 (4)	1 (5)	1 (7)	0	1 (13)	0	1 (10)	1 (25)	0	1 (8)	0	1 (16)	0	1 (8)	0	0	0	1 (67)
31–180	5 (20)	7 (35)	4 (27)	4 (57)	3 (38)	2 (40)	1 (10)	0	3 (30)	4 (33)	2 (29)	1 (26)	3 (30)	3 (25)	0	0	1 (20)	0
181–365	4 (16)	5 (25)	2 (13)	3 (43)	0	3 (60)	0	0	2 (20)	3 (25)	2 (29)	3 (26)	2 (20)	3 (25)	1 (50)	0	0	0
366–548	4 (16)	3 (15)	2 (13)	0	1 (13)	0	2 (20)	1 (25)	1 (10)	2 (17)	1 (14)	1 (11)	1 (10)	2 (17)	0	0	1 (20)	0
549–730	3 (12)	3 (15)	2 (13)	0	0	0	1 (10)	1 (25)	0	2 (17)	0	0	0	2 (17)	0	1 (100)	0	0
731–913	3 (12)	0	2 (13)	0	2 (25)	0	3 (30)	1 (25)	1 (10)	0	0	1 (5)	1 (10)	1 (8)	0	0	0	0
914–1095	2 (8)	1 (5)	1 (7)	0	0	0	0	0	1 (10)	0	0	0	1 (10)		0	0	0	0

E, Endeavor®; I, Resolute Integrity®; ZES, zotarolimus-eluting stent; MACEs, major adverse cardiac events; MI, myocardial infarction; TLR, target lesion revascularization; TVR, target vessel revascularization.

## Discussion

The main findings of this “real-world” all-comers study are as follows: (1) the cumulative incidence of MACEs and ST were comparable between the E-ZES and I-ZES groups after PSM during a 3-year follow-up period. (2) The cumulative incidences of all-cause death, cardiac death, non-fatal MI, any repeat revascularization, TLR, TVR, and non-TVR were not significantly different between the two groups.

From among the five different kinds of ZESs developed by Medtronic Vascular (Santa Rosa, CA, USA) and Abbott Laboratories (Abbott Park, Chicago, IL, USA), the safety of E-ZES and I-ZES was investigated during the long-term follow-up period. I-ZES has been developed more recently and has an advanced stent platform, stent design, and polymer system compared to E-ZES. The B-polymer coating system of I-ZES is composed of three different components, namely hydrophilic C19 component, hydrophobic C10 component, and a water-soluble polyvinyl pyrrolidinone component, and offers potentially improved biocompatibility and extended release of zotarolimus, with 85% of drug the released within 60 days and the remainder getting released over a period up to 180 days [14]. Whereas E-ZES employs a modular technology, I-ZES used the advanced continuous sinusoidal technology, which provides continuous flexibility, smoother tracking, and deliverability [15,16]. Despite these differences, the major clinical outcomes were found to be similar for the two ZESs. Similar outcomes were also reported by Di Santo et al., who assessed the comparative safety and efficacy of R-ZES and I-ZES [2]. R-ZES utilizes Driver® bare-metal stent with a modular design similar to that of E-ZES, and had a PS-adjusted odds ratio (OR) for MACEs of 1.37 (95% CI; 0.46–4.07, *P* = 0.57). In addition, according to Di Santo et al., modifications in the stent platform design do not likely translate into differences in the clinical outcomes. With regard to the polymer system in their study, both the ZESs had the same B-polymer system. However, in the present study, the P-polymer system (E-ZES) and B-polymer system (I-ZES) were compared. In another study, it was suggested that the cumulative incidence of TLR (HR, 0.72; 95% CI, 0.52–1.00; *p* = 0.52) and cardiac death or MI (HR, 1.15; 95% CI, 0.66–2.02; *p* = 0.62) was similar in the E-ZES and R-ZES groups [17]. Considering the results described in the present study and those of previous studies, the type of the polymer does not play any important role in terms of long-term outcomes for patients who underwent PCI with ZESs. However, Iqbal et al. compared the 2-year mortality and TVR in patients with E-ZES and R-ZES [18]. The 2-year mortality (4.1% vs. 6.4%, *p* < 0.001) and TVR (6.8% vs. 10.7%, *p* < 0.001) in the R-ZES group were significantly lower compared to those in the E-ZES group. According to these authors, the newer polymer (B-polymer) was associated with the lower mortality rate and TVR rate. Therefore, we believe that this issue is debatable and further large-scale, randomized, well-controlled trials with longer follow-up would be needed to verify these points. In one meta-analysis, E-ZES was related to increased risk of ischemia-driven TVR (OR, 1.95; 95% CI, 1.40–2.73; *p* < 0.001) when compared with other rapamycin-analogue drug (‘limus’)-eluting stents (LES) [19]. However, the risk of MI (OR, 0.91; 95% CI, 0.54–1.54, *p* = 0.73), cardiac death (OR, 1.02; 95% CI, 0.54–1.91, *p* = 0.96), and ST (OR, 1.10; 95% CI, 0.50–2.44, *p* = 0.81) was similar for E-ZES and LES [20].

ST is another debatable issue in the DES era. In the first month after DES implantation, the polymer plays an important role in inhibiting neointimal hyperplasia by controlling drug-release kinetics [4,21]. Because the B-polymer system has a capacity for longer duration of zotarolimus release, we can expect decreased rates of ST [2]. However, the 3-year ST rates were not significantly different between the two groups in our study (acute ST [0.5% vs. 0.5%, *p* = 1.000], subacute ST [1.0% vs. 0.5%, *p* = 0.562], late ST [0.5% vs. 0.0%, *p* = 0.317], and very late ST [0.5% vs. 0.0%, *p* = 0.317] after PSM. In the TWENTE II trial, the cumulative incidence of definite or probable ST for I-ZES was 1.4% during a 3-year follow-up [22]. In our study, the 3-year overall definite/probable ST rate of ST was 2.6% in the E-ZES and 1.0% in the I-ZES (*p* = 0.449). According to the result of a 5-year follow-up from the ENDEAVOR IV trial, the overall definite/probable ST rate of E-ZES was 1.3% and very late ST for E-ZES was 0.4% [23]. In this study, the three-year overall definite/probable ST rate for E-ZES was 2.6% and very late ST rate of E-ZES was 0.5%. In case of ST, there is no consensus about the relative superiority of E-ZES and I-ZES.

Unexpectedly, there are very limited long-term clinical outcome data comparing the clinical outcomes among the same class of DESs, especially among the different types of stent platform, stent design, and different polymer system in patients who underwent successful PCI. Thus, our results can provide very useful clinical information and trends for E-ZES and I-ZES to some extent, during long-term follow-up periods in the DES era.

There are several limitations to this study. First, this was a non-randomized, single-center study, similar to every ‘‘real-world’’ registry, and there could have been some under-reporting and/or missed data, which might have affected the end results. Second, unfortunately, functional or imaging studies were done only for a small number of patients (<10%) because of cost constraints. In Korea, currently there is no reimbursement program for intravascular ultrasound (IVUS) and optical coherence tomography (OCT) in addition to coronary angiography. Furthermore, the fractional flow reserve (FFR) is partially available under very limited indications during PCI [13]. Hence, we could not perform a fine analysis for pattern and amount of neointimal hyperplasia between the two stents. Third, the strategy of antiplatelet therapies (e.g., DAPT or triple antiplatelet therapy) was left to the physician’s discretion, which might have influenced the major clinical outcomes. Fourth, because this study was a non-randomized, observational, retrospective study, the long-term use of medications was not strictly controlled by the investigators. As a result, the follow-up period and the duration of maintenance of medication could have varied for individual patients. Finally, the cumulative incidence for propensity-matched patients also showed diverging curves for the I-ZES and D-ZES group patients, favoring I-ZES, although it did not reach statistical significance, and might be a function of sample size. This indicates that the lack of difference might be a function of the sample size rather than the difference being truly absent. Therefore, although this study was an all-comers registry, the number of patients enrolled was limited and could have been underpowered to define major clinical outcomes.

## Conclusions

In conclusion, although I-ZES utilizes a more advanced stent platform, stent design, and polymer system than E-ZES, the cumulative incidences of MACE and ST were similar for the two after PSM, during a 3-year follow-up period in this single-center, all-comers registry. In this study, E-ZES and I-ZES are indicated to be equally safe and effective treatment options for significant coronary artery stenosis. However, this result would be more precisely defined by larger study population and long-term follow-up registries, or by undertaking randomized and controlled trials in the future.

## Abbreviations

B-polymer = BioLinx polymer

DES = drug-eluting stent

E-ZES = Endeavor®

I-ZES = Resolute integrity®

MACEs = major adverse cardiac events

P-polymer = phosphorylcholine polymer

PSM = propensity score-matched

R-ZES = Endeavor resolute®

S-ZES = Endeavor sprint®

ZES = zotarolimus-eluting stent
